# Bayesian inference of reassortment networks reveals fitness benefits of reassortment in human influenza viruses

**DOI:** 10.1073/pnas.1918304117

**Published:** 2020-07-06

**Authors:** Nicola F. Müller, Ugnė Stolz, Gytis Dudas, Tanja Stadler, Timothy G. Vaughan

**Affiliations:** ^a^Department of Biosystems Science and Engineering, ETH Zurich, 4058 Basel, Switzerland;; ^b^Swiss Institute of Bioinformatics, 1015 Lausanne, Switzerland;; ^c^Vaccine and Infectious Disease Division, Fred Hutchinson Cancer Research Center, Seattle, WA 98109;; ^d^Department of Biological and Environmental Sciences, University of Gothenburg, SE-40530 Gothenburg, Sweden

**Keywords:** phylogenetics, phylodynamics, infectious diseases, BEAST, MCMC

## Abstract

Reassortment is an important source of genetic diversity in segmented viruses and is the main source of novel pathogenic influenza viruses. Despite this, studying the reassortment process has been constrained by the lack of a coherent, model-based inference framework. Here, we introduce a coalescent-based model that allows us to explicitly model the joint coalescent and reassortment process. In order to perform inference under this model, we present an efficient Markov chain Monte Carlo algorithm to sample rooted networks and the embedding of phylogenetic trees within networks. This algorithm provides the means to jointly infer coalescent and reassortment rates with the reassortment network and the embedding of segments in that network from full-genome sequence data. Studying reassortment patterns of different human influenza datasets, we find large differences in reassortment rates across different human influenza viruses. Additionally, we find that reassortment events predominantly occur on selectively fitter parts of reassortment networks showing that on a population level, reassortment positively contributes to the fitness of human influenza viruses.

## Significance

Genetic recombination processes, such as reassortment, make it complex or impossible to use standard phylogenetic and phylodynamic methods. This is due to the fact that the shared evolutionary history of individuals has to be represented by a phylogenetic network instead of a tree. We therefore require novel approaches that allow us to coherently model these processes and that allow us to perform inference in the presence of such processes. Here, we introduce an approach to infer reassortment networks of segmented viruses using a Markov chain Monte Carlo approach. Our approach allows us to study different aspects of the reassortment process and allows us to show fitness benefits of reassortment events in seasonal human influenza viruses.

Through rapid evolution, human influenza viruses are able to evade host immunity in populations around the globe. In addition to mutation, reassortment of the different physically unlinked segments of influenza genomes provides an important source of viral diversity (1). If a cell is infected by more than one virus, progenitor viruses can carry segments from more than one parent (2). With the exception of accidental release of antigenically lagged human influenza viruses (3), reassortment remains the sole documented mechanism for generating pandemic influenza strains (e.g., refs. 4–6).

To characterize reassortment events, tanglegrams, comparison between tree heights (7, 8), or ancestral state reconstructions (9) are typically deployed. These approaches identify discordance between different segment tree topologies or differences in pairwise distances between isolates across segment trees. Tanglegrams in particular require a substantial amount of subjectivity and have been described as potentially misleading (10).

While the reassortment process has been intensively studied (e.g., refs. 7–9 and 11), there is currently no explicit model-based inference approach available. We address this by introducing a coalescent-based model for the reassortment of viral lineages. In this phylogenetic network model, ancestral lineages carry genome segments, of which only a subset may be ancestral to sampled viral genomes. As in a normal coalescent process, network lineages coalesce (merge) with each other backward in time at a rate inversely proportional to the effective population size. We model reassortment (splitting) events as a result of a constant-rate Poisson process on network lineages. At such a splitting event, the ancestry of segments on the original lineage diverges, with a random subset following each new lineage. We thus explicitly model reassortment networks and the embedding of segment trees within these, allowing us to infer these parameters from available sequence data.

The reassortment process modeled in this way differs from other recombination processes in that it is known where on the genome recombination of genetic material occurs and in that there is no ordering of the segments. The lack of linkage between segments means that at a reassortment event, any subset of segments can originate from either parent.

In order to perform inference under such a model, the reassortment network and the embedding of each segment tree within that network must be jointly inferred. This is similar to the well-known and challenging problem of inferring ancestral recombination graphs (ARGs). While many approaches to inferring ARGs exist, some are restricted to tree-based networks (12, 13), meaning that the networks consist of a base tree where recombination edges always attach to edges on the base tree. Other approaches (e.g., ref. 14) rely on approximations (15) and are not applicable to the reassortment model due to its aforementioned lack of segment ordering. Completely general inference methods exist (16), but these are again not directly applicable to modeling reassortment and furthermore tend to be highly computationally demanding.

Here, we introduce a Markov chain Monte Carlo (MCMC) approach specifically designed to jointly sample reassortment networks and the embedding of segment trees within those networks under the coalescent model, without any additional approximations. This approach allows us to jointly infer the reassortment network, the phylogenetic trees of each segment, the reassortment and coalescent rates, as well as evolutionary rates.

We first show that this approach is able to retrieve reassortment rates, effective population sizes, and reassortment events from simulated data. Secondly, we discuss how a lack of genetic information influences the inference of these parameters. Thirdly, we show how using the coalescent with reassortment can influence the inference of effective population sizes, as well as evolutionary rates. We then apply this approach to quantify reassortment across the five seasonal human influenza subtypes, as listed in *SI Appendix*, Table S1. Finally, we study how reassortment rates differ on edges with high and low fitness of these reassortment networks.

## Results

### Inference of Effective Population Sizes and Reassortment Rates Are Reliably Inferred from Genetic Sequence Data.

In order to test our ability to infer effective population sizes and reassortment rates from genetic sequences, we performed a well-calibrated simulation study (*Simulations*). Using our MCMC approach, we then inferred the reassortment network, segment tree embedding, effective population sizes, and reassortment rates from these genetic sequences.

**Fig. 1.**
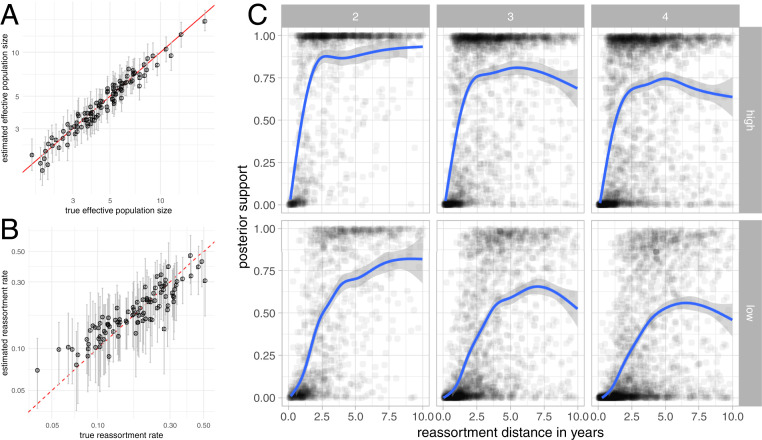
Estimates of effective population sizes and reassortment rates from simulated genetic sequences. (*A*) Estimated effective population sizes and 95% CIs (*y* axis) vs. simulated effective population sizes on the *x* axis. (*B*) Estimated reassortment rates and 95% CI (*y* axis) vs. simulated reassortment rates on the *x* axis. (*C*) Posterior support for true reassortment events (*y* axis) given the reassortment distance (*x* axis). Inference of reassortment networks from sequences simulated with a evolutionary rate of $5\times 10^{-3}$ mutations per site and year (top row) and $5\times 10^{-4}$ mutations per site and year (bottom row). From left to right, the reassortment events are for networks with two, three, and four segments.

Fig. 1 *A* and *B* shows that we are able to correctly retrieve effective population sizes and reassortment rates from simulated genetic sequences. Effective population sizes are estimated more precisely than reassortment rates, which is expected considering that there are typically many more coalescent events in a network than reassortment events. Lower evolutionary rates do not greatly decrease our ability to infer effective population sizes and reassortment rates (*SI Appendix*, Fig. S1). Additionally, these results also hold when using the more complex Hasegawa–Kishino–Yano (HKY) + $\Gamma_{4}$ site model to simulate sequences (*SI Appendix*, Figs. S2 and S3).

To test how well true reassortment events are recovered, we computed the probability of observing exactly the same reassortment events as present in the true (simulated) network. We considered two reassortment events to be the same if the subtree of each segment below that node is the same and if the relative direction of each segment at the reassortment event is exactly the same (*Network Summary*).

As shown in Fig. 1*C*, reassortment events are recovered with good support, particularly with increasing reassortment distance. The reassortment distance denotes how much independent evolution happened on the two parent viruses of the reassortment event (*Reassortment Distance*). This is particularly true when we only look at reassortment events between pairs of segments and drops when we look at three or four segments. This decrease is driven by our definition that two reassortment events are only the same if all segments reassort in the same relative direction at the same time with exactly the same clade below the segment trees: a requirement that becomes harder to satisfy as the number of segments increases. As expected for methods that correctly take into account phylogenetic uncertainty, the posterior support decreases when lower evolutionary rates are used to simulate the sequences of the segments.

### Joint Inference from Full Genomes Increases Precision of Node Ages.

We compared the internal node ages inferred using the coalescent with reassortment to ages inferred under the assumption that all segments evolved independently under the standard coalescent model. To do this, we first compiled datasets of different seasonal human influenza A subtypes, as well as influenza B (details in the *Materials and Methods*). From each of these, we generated 10 datasets consisting of a random sample of sequences. The 10 different datasets were generated in order to prevent individual sequences from biasing the results.

**Fig. 2.**
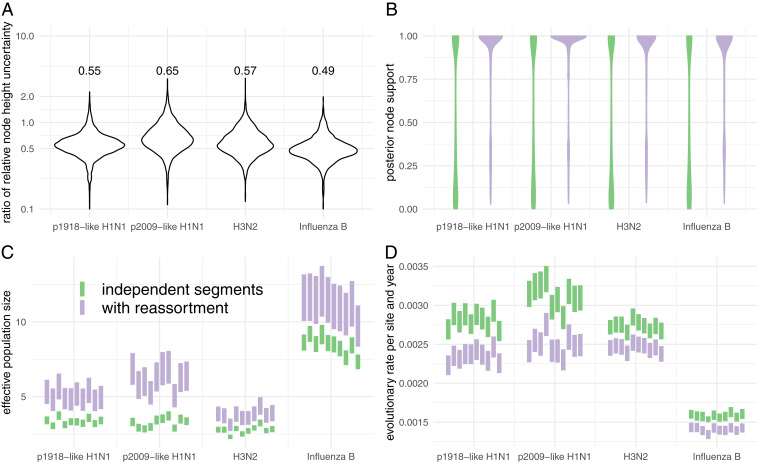
Comparison of estimates between the coalescent with reassortment and assuming that each segment codes for an independent realization of the same coalescent process. (*A*) Comparison of the relative width of the 95% HPD interval of segment tree node heights using 10 random subsets. The vertical axis shows the distribution of ratios of the relative width of the 95% HPD intervals of the coalescent with reassortment over the coalescent assuming independent segment evolution. These values demonstrate a strong reduction in node height uncertainty when using the coalescent with reassortment over the coalescent with independent segments. (*B*) Comparison between the distribution of posterior clade support of segment trees found the MCC segment trees using 10 random subsets. (*C*) Comparison between the inferred effective population sizes. When assuming each segment is an independent realization of the same coalescent process, the effective population sizes are inferred to be much smaller and much more certain. (*D*) Comparison between the inferred clock rates. The coalescent with reassortment infers lower clock rates.

We then analyzed each of these subsampled datasets once using the coalescent with reassortment and once using a normal coalescent prior with shared effective population size across all segments, but assuming that each segment evolved independently. We first computed the 95% highest posterior density (HPD) interval of node heights for each clade that was supported by both approaches with a posterior probability of more than 0.5. We then normalized the difference between the lower and upper bound of the 95% HPD interval, by the median node height estimate to get the relative width of the HPD interval for each clade. As shown in Fig. 2*A*, using the coalescent with reassortment reduces the uncertainty of node height estimates of segment tree nodes by 35% for p2009-like H1N1 up to over 50% for influenza B.

Next, we computed the distribution of clade supports for clades represented in the maximum clade credibility (MCC) trees (as described by ref. 17) inferred using the two approaches. As shown in Fig. 2*B*, segment tree clades are far better resolved when using the coalescent with reassortment for all datasets.

We then compared the effective population sizes and evolutionary rates inferred using the two approaches. The coalescent with reassortment infers higher effective population sizes for all datasets (Fig. 2*C*). This also influences the inferred clock rates, since lower effective population sizes put stronger weight on shorter branches and therefore larger clock rates (Fig. 2*C*). We explain this discrepancy as follows. While we currently do not account for population structure, the datasets we analyze are in part shaped by population structure, such as geographic structure. Ignoring this structure likely affects the coalescent with reassortment differently compared with assuming independent segments. Coalescent events closer to the tips are more likely between lineages that are, for example, geographically more closely related and can be assumed to occur rapidly and provide information about low effective population size values. Coalescent events deeper in the tree on the other hand are more representative of those between geographically more separated lineages. These events therefore provide information about larger effective population sizes. Coalescent events across different segments that occur close to the tips are less likely to have encountered reassortment events. In the coalescent with reassortment, they are therefore interpreted as one event, whereas in the coalescent with independent segments, they are interpreted as eight. Coalescent events deeper in the tree are more likely between lineages that encountered reassortment events and are therefore more likely to provide independent information about the population process. The coalescent with independent segments assumes that all coalescent events provide the same amount of information about the population process and will consequently favor information about the population process closer to the tips. This leads to differences in the estimated effective population sizes, which then leads to differences in the estimated clock rates.

We also compared the performance of the two approaches by inferring tip dates (18). The tips (leaf nodes) are the only nodes in the trees or network for which we can actually presume to know the true age, which is set by the sample collection time. To compare the two approaches, we compiled 1,000 smaller influenza A/H3N2 datasets, each composed of 20 genomes. Of those datasets, 500 were randomly sampled from an random interval of 2 y between 1995 and 2019. The remaining 500 datasets were assembled using a random sampling interval of 10 y between 1995 and 2019. From each of these datasets, we randomly selected a single genome and inferred its sampling time using both approaches, conditional on the sampling times of the remaining genomes.

The 95% HPD of the sample time posteriors under the coalescent with reassortment contains the true sampling time interval in 91% of cases for the 2-y sampling interval and in 89% for the 10-y sampling interval (*SI Appendix*, Fig. S4). On the other hand, the 95% HPD of the sample time posteriors generated by the independent segment coalescent model contains the true sampling time in only 68% (2-y sampling interval) and 77% (10-y sampling interval) of cases.

### Contrasting Reassortment Rates across Different Human Influenza Viruses.

We compared the reassortment rates of the five different seasonal human influenza types listed in *SI Appendix*, Table S1. These include the same datasets described in *Joint Inference from Full Genomes Increases Precision of Node Ages*, as well as an influenza A/H2N2 dataset sampled between 1957 and 1970. We then jointly inferred the reassortment network, the embedding of segment trees, evolutionary rates, effective population sizes, and reassortment rates of these viruses. We find that the estimated reassortment rates vary greatly between different influenza viruses.

**Fig. 3.**
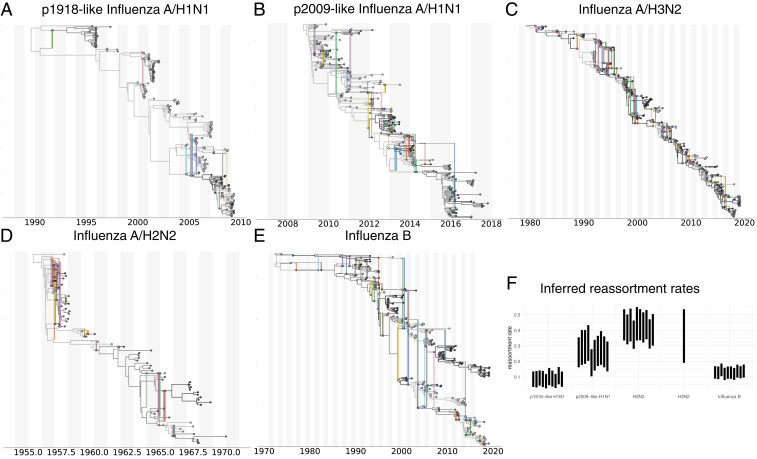
Estimates of MCC networks and reassortment rates of different human influenza viruses. MCC networks of p1918-like influenza A/H1N1 (*A*), p2009-like influenza A/H1N1 (*B*), influenza A/H2N2 (*C*), influenza A/H3N2 (*D*), and influenza B (*E*). These MCC networks are shown for 1 of the 10 random subsets. The MCC networks of all random subsets are shown in *SI Appendix* , Figs. S5–S8. (*F*) Here, we show the inferred reassortment rates (*y* axis) for different influenza viruses on the *x* axis. The reassortment rates are per lineage and year.

Influenza A/H3N2 shows the highest rates of reassortment, while pandemic 1918 (p1918)-like H1N1 and influenza B show the lowest inferred rates of reassortment (Fig. 3). H2N2 and 2009 pandemic (p2009)-like H1N1 show intermediate rates of reassortment, although the uncertainty on those estimates is quite large.

Differences between p1918-like H1N1, H2N2, and H3N2 are particularly interesting since these strains share many common segments. All segments with the exception of HA, NA, and PB1 of influenza A/H3N2 originate from the p1918-like H1N1 strain (19), and H2N2 and H3N2 only differ in HA and PB1. p2009-like human H1N1, which became seasonal in the years after the 2009 pandemic, on the other hand, has one segment (PB1) that originates from human H3N2 and three segments (HA, NP, and NS) derived from classic swine viruses that are descended from a p1918-like strain (5). It shows similar reassortment rates to H3N2 but highly elevated levels compared with the p1918-like H1N1 strain.

Such variations in reassortment rates may be driven by a number of factors. Differences in coinfection rate (which may be linked to the effective population size) lead to different probabilities of viruses being in the same host at the same time and therefore to a difference in the rate at which reassortants appear. In particular, the higher incidence of influenza A/H3N2 and the correspondingly likely higher number of coinfection events compared with other influenza A viruses or influenza B viruses may contribute to the higher observed reassortment rate in that case. Additionally, reassortants in the different seasonal human influenza viruses could have, on average, a different fitness and therefore be more or less likely to be transmitted.

### Reassortment Events Occur on Fitter Parts of Reassortment Networks.

Next, we test if there is a fitness effect associated with reassortment events. To do so, we classify every network edge from the posterior distribution of inferred networks as either “fit” or “unfit.” We define a fit edge to be any edge having descendants that still persist at least 2 y into the future, while every other edge in the network is defined to be unfit. Unfit here, however, still means that the viruses are likely fit enough to be transmitted. If reassortment events are beneficial, lineages that are the result of reassortment events should have a higher survival probability and are therefore more likely to persist further into the future.

**Fig. 4.**
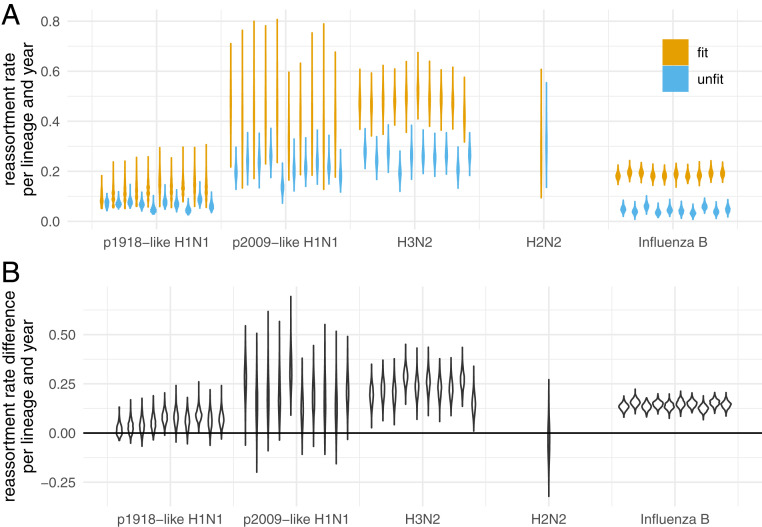
Estimates of reassortment rates on fit and unfit edges. (*A*) Here, we show the number of reassortment events on fit and unfit edges of the networks divided by the total length of fit and unfit edges. Fit edges are defined as having a sampled descendant at least 2 y into the future. Every other edge is considered unfit. These rates are shown for different human influenza viruses on the *x* axis. The violin plots denote the distribution of theses ratios over the posterior distribution of networks. (*B*) Here, we show the difference between fit and unfit reassortment rates. Values above 0 indicate that reassortment events are more likely to occur on fitter, while values below 0 indicate that reassortment events are more likely to occur less fit edges.

To test if reassortment is beneficial, we calculated the number of reassortment events on fit edges and on unfit edges for all networks in the posterior distribution of the MCMC. We then divided this number by the total length of fit respectively unfit edges. As shown in Fig. 4, reassortment events occur at a higher rate on fit edges of the H3N2 and influenza B networks than they do on unfit edges. This suggests that reassortment is beneficial to the fitness of influenza A/H3N2 and influenza B viruses.

For the other human influenza viruses, fitness benefits of reassortment are less pronounced. For p2009-like H1N1 and H2N2, the sampling time windows (both) or number of samples (H2N2) was however rather small, and the results are likely driven by a lack of data. p1918-like H1N1 on the other hand had relatively few reassortment events, overall driven by a low reassortment rate, and the results are likely driven by a lack of reassortment events. These same general patterns hold when the definition of what is a fit edge is changed to having descendants at least 4 or 6 y into the future (*SI Appendix*, Figs. S9 and S10). For p2009-like H1N1, where the overall sampling interval is only 10 y, the evidence for fitness benefits of reassortment events, however, decreases when using 4 and 6 y instead of 2 y.

We conducted further analysis to probe the robustness of our result for H3N2, for which densely sampled sequences of long time intervals are available. We analyzed two more influenza A/H3N2 datasets, one sampled between 1980 and 2005 and one sampled from 2005 until the present. For both datasets, we find higher rates of reassortment on fit edges (*SI Appendix*, Fig. S11). Over shorter time frames, the effect of fitness is less visible since selection has not had enough time to filter out less fit strains. In turn, this means that if we look at datasets sampled over short times (for example, over 2 y), we should estimate reassortment rates consistent with the estimated rates on unfit edges. To test this, we compiled 9 datasets, each with 100 to 200 sequences sampled from 2 seasons between 2000 and 2018. Averaged over all nine datasets, we find the short-term reassortment rate to be approximately 0.2 reassortment events per lineage per year, which is consistent with the reassortment rate estimates for unfit edges (*SI Appendix*, Fig. S12).

Finally, we sought to rule out the possibility that these patterns are simply a property of our reassortment model. To do this, we simulated networks under the coalescent with reassortment with the reassortment rates and effective population sizes fixed to the mean values estimated from the empirical data and the network tip times fixed to those from the same data. In these simulated networks, each edge has the same fitness. We then recomputed the same fit/unfit reassortment rate statistics from these simulated networks (*SI Appendix*, Fig. S13) and found that the patterns we observed in the empirical data completely disappeared. This strongly suggests that the elevated rate of reassortment on fit lineages is not due to the particulars of our model but is instead a real effect.

## Conclusion

We here present a Bayesian approach to jointly infer the reassortment network, the embedding of segment trees, and the corresponding evolutionary parameters. We show that this approach is able to retrieve reassortment rates, effective population size, and reassortment events from simulated data.

We have used this approach to show that there are large differences in the rates of reassortment across different influenza viruses and that reassortment events occur predominantly in fitter parts of the corresponding reassortment networks. We propose that this is due to selection favoring lineages that have reassorted. Although we have deployed a relatively simple way of defining which edges of a network are fit and which are not, future approaches could more directly incorporate fitness models into these network-type approaches (20, 21). Additionally, our ability to infer which segments coreassort will allow us to further investigate if coreassortment between specific segments drive these fitness benefits.

Even if one is not directly interested in reassortment patterns, our approach allows phylogenetic and phylodynamic inferences to exploit full-genome sequences for reassorting viruses. This helps to avoid bias and increase precision compared with, for instance, assuming segments evolve completely independently. However, a lot of development remains to be done in the direction of incorporating skyline models for population size dynamics (22, 23) together with extending the model to account for population structure (24, 25).

In summary, this approach allows us to perform network inference by directly accounting for a special kind of recombination process, i.e., reassortment. In the future, we will pursue the development of related approaches to account for a variety of other recombination processes.

## Materials and Methods

### The Coalescent with Reassortment.

**Fig. 5.**
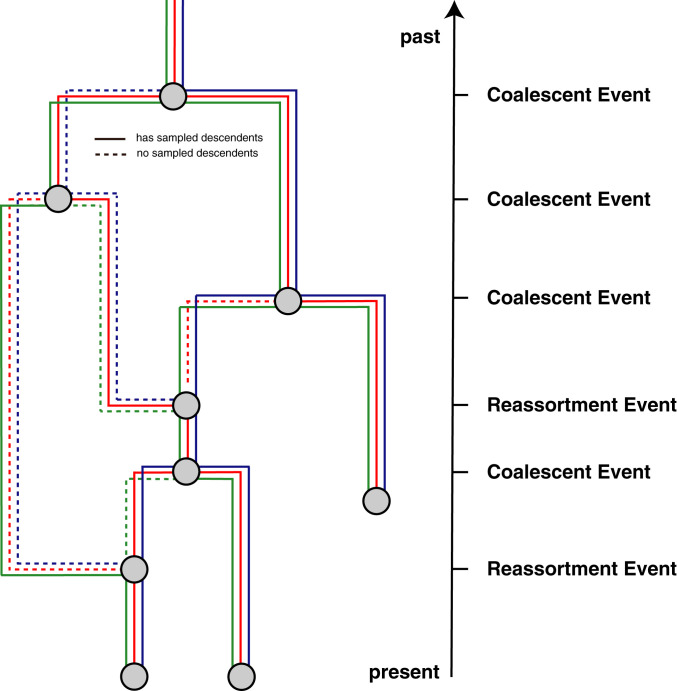
Example reassortment network. Here, we give an example of a reassortment network where we track three different segments differentiated by the different colors through the network. Dashed lines denote segment lineages that do not have sampled descendants. As done in coalescent approaches, we track the network from the present backward in time to the past.

Here, we introduce a model to describe a coalescent process with reassortment. To do so, we define t to be the time (increasing into the past) before the most recent sample and $L_{t}$ as the set of network lineages extant at time t (Fig. 5). Each extant network lineage $l\in L_{t}$ carries the full set of genome segments, S. In general, however, only a subset C(l)⊆S of these are directly ancestral to sampled viruses. We refer to this subset as the “carrying load.” We further define the total number of segments |S| and the number of ancestral segments |C(l)|.

The coalescent with reassortment is a continuous time Markov process that proceeds backward in time. It involves three possible events: sampling, coalescent, and reassortment events. As is usually the case for coalescent approaches, we condition on sampling events. These happen at predefined times and simply increase the number of extant network lineages by 1. Coalescent events occur between two network lineages l and $l^{\prime }$ at a rate that is inversely proportional to the effective population size $N_{e}$ and reduce the number of active network lineages by 1. The smaller the effective population size, the more likely two lineages are to share a recent common ancestor, i.e., the more likely they are to coalesce. Upon a coalescent event, the ancestral segments that the parent lineage p of lineages l and $l^{\prime }$ carries is the union of those carried by l and $l^{\prime }$, i.e.:

$$C\left(p\right)=C\left(l\right)\cup C\left(l^{\prime }\right).$$

This coalescent event in the network only corresponds to a coalescent event in a segment tree when the corresponding segment is present in both C(l) and $C\left(l^{\prime }\right)$.

Reassortment events happen at a rate ρ per lineage per unit time. A reassortment event on lineage l will increase the number of network lineages by 1. The segments carried by lineage l are randomly assigned to the two parent lineages $p_{1}$ and $p_{2}$. This means that the probability of the ancestry of a given segment to follow $p_{1}$, for example, is 0.5.

As we are not interested in the history of segments that are not ancestral to our sample, we explicitly integrate over this ancestry in our model. As with standard coalescent with recombination models, this is done by omitting nonancestral events from the process and modifying the reassortment rate to exactly account for this omission. In our model, the events that are omitted are “reassortment” events on l in which the ancestry of every ancestral lineage in C(l) is assigned to the same parent. (Thus no true reassortment occurs.) Since each segment chooses its parent edge uniformly at random, the probability of either $p_{1}$ or $p_{2}$ being chosen as ancestral to all segments is

$$P\left(C\left(p_{1}\right)=\emptyset \vee C\left(p_{2}\right)=\emptyset \right)=2\times {\left(\frac{1}{2}\right)}^{\left|C\left(l\right)\right|}=f\left(l\right).$$

The effective rate of “observable” reassortments on lineage l is then simply ρ(1−f(l)).

### Calculating the Posterior Probability.

In order to perform joint Bayesian inference of reassortment networks together with the parameters of the associated models, we use a MCMC algorithm to characterize the joint posterior density.

$$P\left(N,\vec{\mu },\theta ,\rho |\vec{D}\right)=\frac{P\left(\vec{D}|N,\vec{\mu }\right)P\left(N|\theta ,\rho \right)P\left(\vec{\mu },\theta ,\rho \right)}{P\left(\vec{D}\right)}.$$ [1]

Here, N represents the full reassortment network (including the embedding of the segment trees); the elements of the vectors $\vec{D}$ and $\vec{\mu }$ represent the segment-specific multiple sequence alignments and their associated molecular substitution models and parameters. The parameters θ and ρ are the effective population size and per-lineage reassortment rate.

The terms on the right-hand side of Eq. **1** include the network likelihood $P\left(\vec{D}|N,\vec{\mu }\right)$, the network prior P(N|ρ,θ), and the joint parameter prior $P\left(\vec{\mu },\theta ,\rho \right)$. Each of these terms is discussed below. [The denominator $P\left(\vec{D}\right)$ is the marginal likelihood of the model and does not concern us here.]

### The Network Likelihood.

The usual conditional independence of sites assumption made in phylogenetic analyses allows us to factorize the network likelihood in terms of the individual segment tree likelihoods:

$$P\left(\vec{D}|N,\vec{\mu }\right)=\underset{s\in S}{\prod }P\left(D_{s}|T_{s},\mu_{s}\right).$$

These tree likelihoods can be computed using the standard pruning algorithm (26).

### The Network Prior.

The term P(N|θ,ρ) denotes the probability of the network and the embedding of segment trees under the coalescent with reassortment model, with effective population size θ and per-lineage reassortment rate ρ. It plays the role of the tree prior in standard phylodynamic analyses.

We can calculate P(N|θ,ρ) by expressing it as the product of exponential waiting times between events (i.e., reassortment, coalescent, and sampling events):

$$P\left(N|\theta ,\rho \right)=\overset{\#events}{\underset{i=1}{\prod }}P\left({event}_{i}|L_{i},\theta ,\rho \right)\times P\left({interval}_{i}|L_{i},\theta ,\rho \right),$$

where we define $t_{i}$ to be the time of the $i^{th}$ event and $L_{i}$ to be the set of lineages extant immediately prior to this event. [That is, $L_{i}=L_{t}$ for $t\in \left[t_{i-1},t_{i}\right)$.]

### Event Contribution.

The event contribution of the *i*th event in the network is different depending on if the *i*th event is a coalescent or reassortment event. If the *i*th event is a coalescent event between lineage $l_{1}$ and $l_{2}$, the event contribution is the probability density of this particular pair of lineages coalescing at time $t_{i}$. For a constant-sized coalescent model, this is

$$P\left({event}_{i}|L_{i},\theta ,\rho \right)=\frac{1}{\theta }.$$

On the other hand, if the *i*th event is a reassortment event on lineage l, the event contribution is the probability density of an (observable) reassortment event to occur on that lineage, i.e.:

$$P\left({event}_{i}|L_{i},\theta ,\rho \right)=\rho \left[1-2\times {\left(\frac{1}{2}\right)}^{\left|C\left(l\right)\right|}\right].$$

As we condition on sampling events, their event contribution is always simply 1.

### Interval Contribution.

The interval contribution $P\left({interval}_{i}|L_{i},\theta ,\rho \right)$ is the probability of not observing any event in a given time interval. Three different types of events can happen in the coalescent with reassortment: sampling, coalescent, and reassortment events. Since we condition on the times of the sampling events, only coalescent and reassortment events are produced. Given the total rate $\Lambda_{i}$ (probability per unit time) with which these occur in the interval immediately prior to event i, the interval contribution can be written as:

$$P\left({interval}_{i}|L_{i},\theta ,\rho \right)=\exp\left[-\Lambda_{i}\left(t_{i}-t_{i-1}\right)\right].$$

The total rate is the sum of the coalescence rate $\lambda_{i}^{\left(c\right)}$ and the reassortment rate $\lambda_{i}^{\left(r\right)}$. The coalescence rate depends on the number of lineages extant at a particular time and the effective population size in the usual way:

λi(c)=|Li|21θ.

The rate of observable reassortment events is:

$$\lambda_{i}^{\left(r\right)}=\rho \left[|L_{i}|-\underset{l\in L_{i}}{\sum }{\left(\frac{1}{2}\right)}^{|C\left(l\right)|-1}\right].$$

Note that $\lambda_{i}^{\left(r\right)}$ is generally less than the total rate of reassortment events in this interval, which would be simply $\rho |L_{i}|$, as, i.e., $\lambda_{i}^{\left(r\right)}$ excludes reassortment events that produce lineages carrying no ancestral segments.

### The Parameter Priors.

The term $P\left(\vec{\mu },\theta ,\rho \right)$ denotes the joint prior distribution of all model parameters. We factorize the prior distribution by writing it as the product of the individual parameter priors $P\left(\vec{\mu }\right)$, P(θ), and P(ρ). This asserts that our prior information on any one of these model parameters is independent of the prior information we have for the others.

### An MCMC Algorithm for Reassortment Networks.

In order to perform MCMC sampling of network and the embedding of segment trees within these networks, we introduce several MCMC operators (i.e., proposal distributions). These operators often have analogs in operators used to explore different phylogenetic trees. Here, we briefly summarize each of these operators, providing the complete details in the supplement.

#### Add/remove operator.

The add/remove operator adds and removes reassortment events. An extension of the subtree prune and regraft move for networks (27) to jointly operate on segment trees as well.

#### Segment diversion operator.

The segment diversion operator changes the path segments take at reassortment events.

#### Exchange operator.

The exchange operator changes the attachment of edges in the network while keeping the network length constant.

#### Subnetwork slide operator.

The subnetwork slide operator changes the height of nodes in the network while allowing to change the topology.

#### Scale operator.

The scale operator scales the heights of individual nodes or the whole network without changing the network topology.

#### Gibbs operator.

The Gibbs operator efficiently samples any part of the network that is older than the segment tree roots and is thus not informed by any genetic data.

#### Empty segment preoperator.

The empty segment preoperator augments the network with edges that do not carry any segments for the duration of a move, to allow larger jumps in network space.

We validate the implementation of the coalescent with reassortment network prior as well as all operators in the supplement.

### Summarizing Reassortment Networks.

To summarize a distribution of networks, we use a similar strategy to the MCC strategy (as described by ref. 17) used by phylogenetic inference software such as BEAST to summarize distributions of trees. We first compute all unique coalescent and reassortment nodes that were encountered during the MCMC. To do so requires that we define when two coalescent or reassortment nodes are the same. We define two coalescent nodes to be the same if 1) the parent edges of those nodes carry the same segments and 2) if the subtree below each segment includes exactly the same clades between the two coalescent nodes. We define two reassortment nodes to be the same if 1) both parent edges carry the same segments in the same relative orientation and 2) if the subtree below each segment includes exactly the same clades between the two reassortment events. A side effect of this definition is that the more segments we include in the summary, the more likely two nodes will be considered different nodes.

While the number of coalescent and reassortment nodes in the network changes over the course of the MCMC, the number of coalescent nodes on the segment trees is constant. In order to avoid dimensionality issues when summarizing, we first compute the frequency of observing each coalescent node over the course of the MCMC. We then weight this frequency by the number of coalescent events on segment trees this coalescent node corresponds to. We next choose the network that maximizes those weighted clade credibilities as the MCC network.

In order to compute the posterior support of each reassortment event in the MCC network, we next compute the frequency of observing each reassortment event in the MCC network during the MCMC.

Since we require the network to be rooted, we track segments even after the root of a segment tree is reached. These patterns are however not supported by any genetic information and follow the prior distribution only. For the summary of networks, we therefore remove segments from edges if the root of a segment tree has been reached. Additionally, we remove reassortment loops, i.e., events that start on one edge and then directly reattach to the same edge. Since the support for individual events can greatly depend on how many segments are analyzed, we have also implemented an option to only summarize over a subset of the segments, while ignoring others.

### Reassortment Distance.

For any reassortment event where segments a and b take different paths, follow segment a until it reaches a network edge that carries segment b. We repeat this for any pair of segments that took different paths to get the common ancestor height between any two segments. We then define the reassortment distance and a reassortment event as the minimal difference between the height of the reassortment event and the common ancestor height of any pair of segments. This seeks to denote for how long segments in the two parent viruses at the reassortment event evolved independently.

### Implementation.

We implemented the MCMC framework for the coalescent with reassortment as a BEAST2 package called CoalRe. This package includes the classes to do simulation and inference under the coalescent with reassortment. The implementation is such that the tree likelihood calculations are separate from the network framework, which allows using the various different site and clock models implemented in BEAST2. Additionally, it can be used with other Bayesian approaches such as nested sampling or parallel tempering. Further, model comparison as well as integration over evolutionary models can be performed. The package can be downloaded by using the package manager in BEAUti. The source code for the software package can be found here: https://github.com/nicfel/CoalRe. A tutorial on how to set up an analysis using the coalescent with reassortment is available at https://taming-the-beast.org/tutorials/Reassortment-Tutorial (28). Networks are logged in the extended Newick format (29) and can be visualized using, for example, https://icytree.org/ (30). Additionally, we provide python scripts to plot networks based on https://github.com/evogytis/baltic.

### Simulations.

In the simulation study, we simulated reassortment networks, using the structured coalescent with randomly drawn effective population sizes and reassortment rates. To do so, we first sampled random effective population sizes from a log normal distribution (mean: 5; SD: 0.5) and reassortment rates from another log normal distribution (mean: 0.2; SD: 0.5). An average effective population size of 5 is similar to the estimated effective population sizes of seasonal human influenza viruses (Fig. 2*C*), and an average reassortment rate of 0.2 per lineage per year is similar to the later estimated reassortment rates for seasonal human influenza viruses (Fig. 3). We then randomly sampled the sampling times of 100 taxa, each with 4 segments, from a uniform distribution between 0 and 20. We simulated reassortment networks alongside the embedding of the segment trees using these parameters. For each segment tree, we next simulated genetic sequences by using the Jukes–Cantor substitution model (31) with an evolutionary rate of $5\times 10^{-3}$ per site and year. We used the Jukes–Cantor model in order to limit the uncertainty coming from the substitution model, allowing us to test the performance of the coalescent with reassortment under idealized conditions. Each segment consisted of 1,000 independently evolving nucleotides. In order to study the effect of reducing the amount of genetic information, we additionally considered the scenario where all segments had the slower evolutionary rate of $5\times 10^{-4}$ per site and year. These two evolutionary rates are higher ($5\times 10^{-3}$) and lower ($5\times 10^{-4}$) than those estimated for seasonal human influenza viruses (Fig. 2*C*).

### Sequence Data Availability and Analysis.

We compiled datasets from several influenza viruses using sequence data downloaded from the Influenza Research Database (https://www.fludb.org) (pandemic and seasonal H1N1, H3N2, and influenza B). For the influenza A/H2N2 dataset, we used the same sequences as in ref. 32. We obtained these sequences from GISAID (https://gisaid.org; acknowledgments table can be found at https://github.com/nicfel/Reassortment-Material/blob/master/Applications/H2N2/oridata/gisaid_acknowledge_table.xls). For all datasets but the influenza A/H2N2 dataset, we used subsampling to produce a final set containing at least 500 samples with an even temporal distribution. We aligned all segments using Muscle 3.8.31 (33).

We then analyzed every influenza virus under the coalescent with reassortment in BEAST 2.5.2 (34) using parallel tempering (35, 36). We assumed the sequences to have evolved under an HKY + $\Gamma_{4}$ model (37, 38), allowing the first two codon positions and the third having different rates (39). This model has been regularly used in previous phylogenetic analyses of seasonal human influenza viruses (see, for example, refs. 40 and 41).

We then jointly estimated all evolutionary rates, the reassortment networks, and embedding of segments trees, as well as the reassortment rates and effective population sizes. For the influenza A/H2N2 dataset, we additionally estimated the sampling times for all sequences for which only the year in which the sample was taken was known. We assessed convergence using effective sample sizes and potential scale-reduction factors (42) computed using coda (43) (*SI Appendix*, Fig. S14).

For virus types with sequences downloaded from the Influenza Research Database (https://www.fludb.org), the full extensible markup language (XML) files to run the datasets are available online. For the influenza A/H2N2 sequences that were obtained from GISAID, we removed the sequence characters from the XML files in order to comply with the relevant license regulations, leaving the accession numbers intact, and thus still allowing reproduction of results based on these data. All other data, such as log files of BEAST2 runs, as well as scripts to analyze and plot results, are available at https://github.com/nicfel/Reassortment-Material.

## Supplementary Material

Supplementary File
